# Clinical Utility of the Candy-Plug Technique Using an Excluder Aortic Extender

**DOI:** 10.3400/avd.oa.21-00018

**Published:** 2021-06-25

**Authors:** Yukihisa Ogawa, Hiroshi Nishimaki, Kiyoshi Chiba, Tomotaka Iraha, Takaaki Maruhashi, Yuka Sakurai, Takeshi Miyairi, Yasuo Nakajima

**Affiliations:** 1 Department of Radiology, St. Marianna University, School of Medicine, Kawasaki, Kanagawa, Japan; 2 Department of Cardiovascular Surgery, St. Marianna University, School of Medicine, Kawasaki, Kanagawa, Japan; 3 Department of Emergency and Critical Care Medicine, Kitasato University School of Medicine, Sagamihara, Kanagawa, Japan

**Keywords:** aortic dissection, rupture, thoracic endovascular aortic repair, candy-plug technique, Excluder aortic extender

## Abstract

**Objective**: To describe the clinical utility and technical aspects of the candy-plug technique using an Excluder aortic extender (Ex-cuff) for false lumen (FL) occlusion in chronic aortic dissection.

**Materials and Methods**: This is a retrospective study analyzing seven consecutive patients (mean age, 63 years; range, 44–78 years; 6 men) with aneurysmal dilatation or rupture in chronic aortic dissection. All patients had undergone thoracic endovascular aortic repair with FL occlusion using this technique. We assessed technical (deployment accuracy) and clinical (no FL backflow on the latest contrast-enhanced computed tomography) success.

**Results**: Technical success was obtained in six patients (86%). Technical failure was caused by the malposition of the candy-plug. The mean follow-up period was 593 days (range, 222–1225 days). Clinical success was obtained in four (57%), and incomplete Amplatzer Vascular Plug (AVP) embolization was seen in two. There was no enlarged FL after the procedure, and all patients are alive during the follow-up periods.

**Conclusion**: The candy-plug technique using an Ex-cuff may be a feasible option; however, it takes time to achieve complete AVP embolization. Therefore, using additional embolic materials should be considered when we use it for the rupture case. (This is a translation of Jpn J Endovasc Interv 2018; 19: 29–35.)

## Introduction

Major complications of chronic type B aortic dissection (cTBAD) include false lumen (FL) dilatation and (impending) rupture. Approximately 35% of patients with cTBAD develop FL dilatation even after optimal medical management.^1)^ The estimated FL rupture rate is 30% once aortic diameter reaches 60 mm. These aortic changes result in a 5-year survival rate of 50%–80%.^2)^

According to guidelines from the European Society of Cardiology, thoracic endovascular aortic repair (TEVAR) is recommended as a first-line treatment in complicated cTBAD cases.^3)^ However, even after TEVAR to close primary entry tear, FL perfusion persists because of distal entry tears in 20%–30% of patients. This persistent FL flow can cause FL rupture.^4)^

The candy-plug technique for FL occlusion was described in 2013.^5)^ Although Kölbel et al. used a combination of the Zenith TX2® (Cook Medical, Bjæverskov, Denmark) and Amplatzer™ Vascular Plug (AVP; St. Jude Medical, Minneapolis, MN, USA), we examined the suitability of the candy-plug technique using an Excluder aortic extender (Ex-cuff; W. L. Gore & Associates, Inc., Flagstaff, AZ, USA).^6)^

We evaluated the clinical outcomes of the candy-plug technique using an Ex-cuff and investigated whether this method would work in the setting of emergency FL rupture.

## Materials and Methods

This study was approved by the Institutional Review Board of St. Marianna University School of Medicine (approval number 5134).

Between July 2014 and July 2017, a total of seven patients (6 male) with cTBAD (mean age, 63 years) were treated by TEVAR and the candy-plug technique with an Ex-cuff.

Two patients had postoperative type A dissection, six patients had an enlarged aneurysm (maximum short-axis diameter, ≥55 mm) or rapid expansion (≥5 mm over 6 months), and one patient had FL rupture. FL rupture was caused by descending graft anastomotic rupture followed by postoperative type A dissection with double-barrel descending aortic repair.

The mean cTBAD treatment duration after onset was 3.8 years (range, 1–9 years), and the mean maximum short-axis diameter was 58.7 mm (range, 44–71 mm). Patient characteristics are summarized in **Table 1**.

**Table 1:** Table 1 Patient demographics and lesion characteristics

Patient number	N=7
Mean age (years) (range)	63 (44–78)
Sex (M : F)	6 : 1
Diagnosis	Post type A (2)
	Chronic type B (5)
Indication of treatment	Aneurysmal dilatation (6)
	Rupture (1)
Median time after onset to treatment of TEVAR with candy-plug technique (years)	3.8 (1–9)
Mean maximum diameter of the aorta (mm)	58.7 (44–71)

The patient inclusion criteria and requirements for the candy-plug technique are as follows: 1) residual FL flow that persisted even after TEVAR closure of the primary entry tear, 2) enable approaching the FL, and 3) a mean FL diameter of ≤34 mm above the distal entry that enabled the candy plug to be deployed.

The study endpoints were technical success rate, clinical success rate, change in aneurysm size, complications, and survival rate.

Technical success was defined as successful TEVAR and accurate candy-plug deployment without complications. Clinical success was defined as complete FL thrombosis above the candy plug on contrast-enhanced computed tomography (CECT). A change in aneurysm size of ≥5 mm was classed as significant.

### Candy-plug preparation using the Ex-cuff (**Fig. 1**)

**Figure 1:**
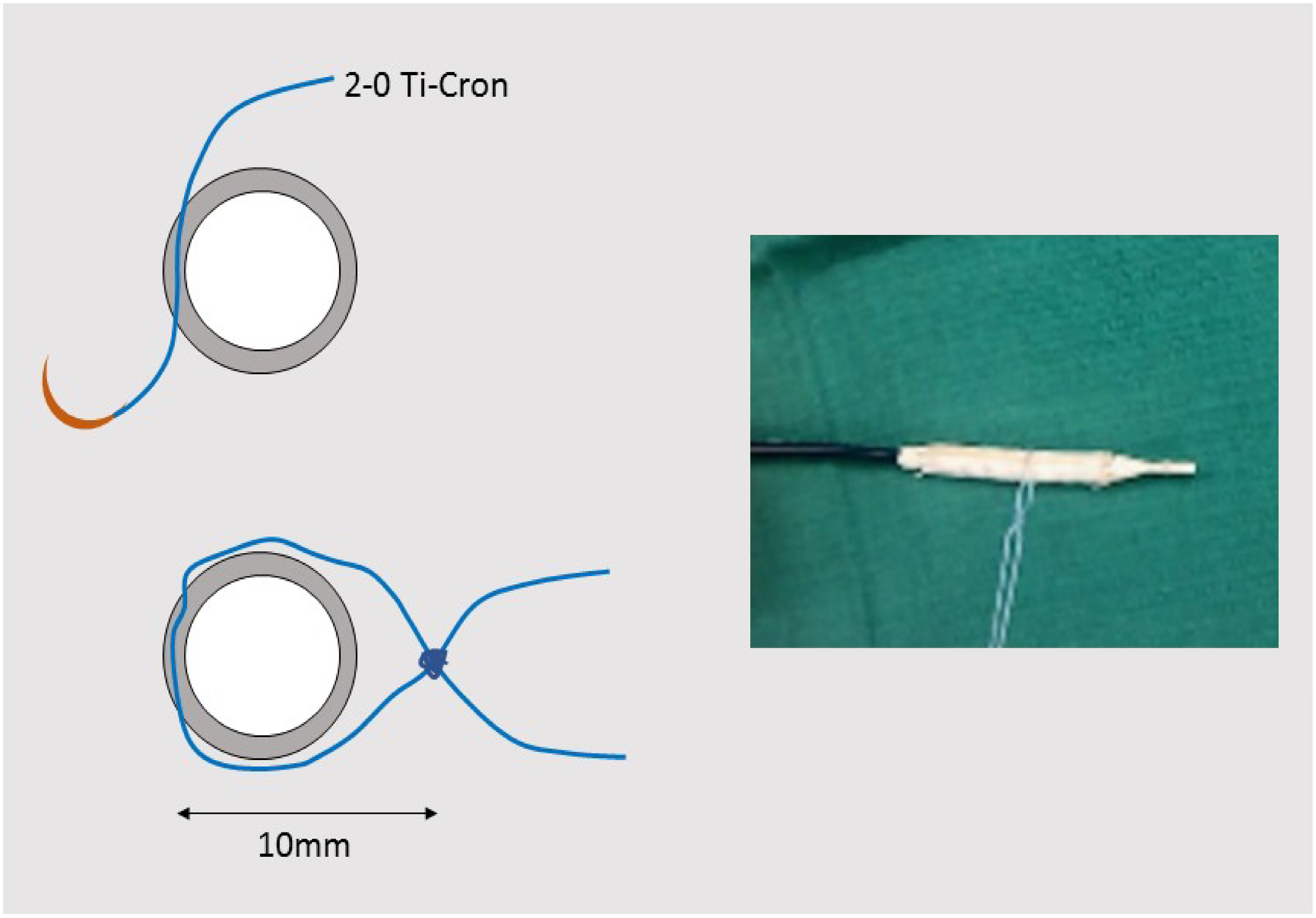
Fig. 1 Preparation of a candy plug using the Ex-cuff. The needle of the Ti-Cron suture was inserted through the Ex-cuff to secure the thread. Complete ligation was achieved with approximately 10 mm of clearance.

Ex-cuff: Excluder aortic extender

A polyester suture (Ti-Cron™; Medtronic, Minneapolis, MN, USA) was inserted through the center or upper one-third of the Ex-cuff to secure the thread and drawn around the Ex-cuff to form a diameter-reducing tie. Complete ligation was achieved with a clearance of approximately 10 mm. A 2F oversized delivery sheath is required to allow the suture stent-graft insertion; thus, a 20-F sheath was required when a 36-mm Ex-cuff was used. A 16-mm AVP I was deployed in the waist of the Ex-cuff to complete occlusion.

### Candy-plug sizing (**Fig. 2**)

**Figure 2:**
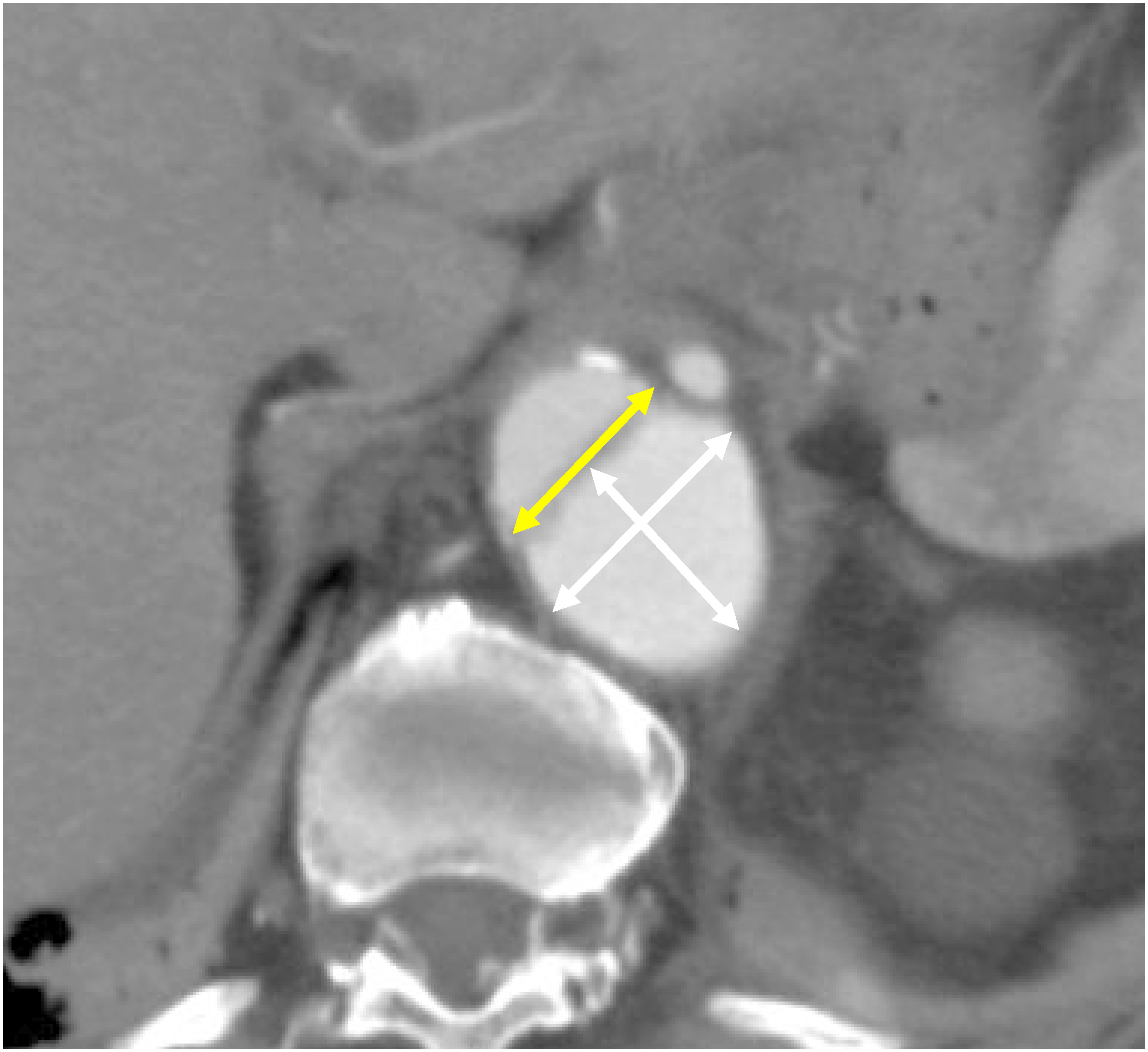
Fig. 2 Candy-plug sizing. TL stent graft≒crescent length (yellow line). Ex-cuff=mean FL diameter (white lines)+10%–20%.

TL: true lumen; FL: false lumen; Ex-cuff: Excluder aortic extender

**Figure 3:**
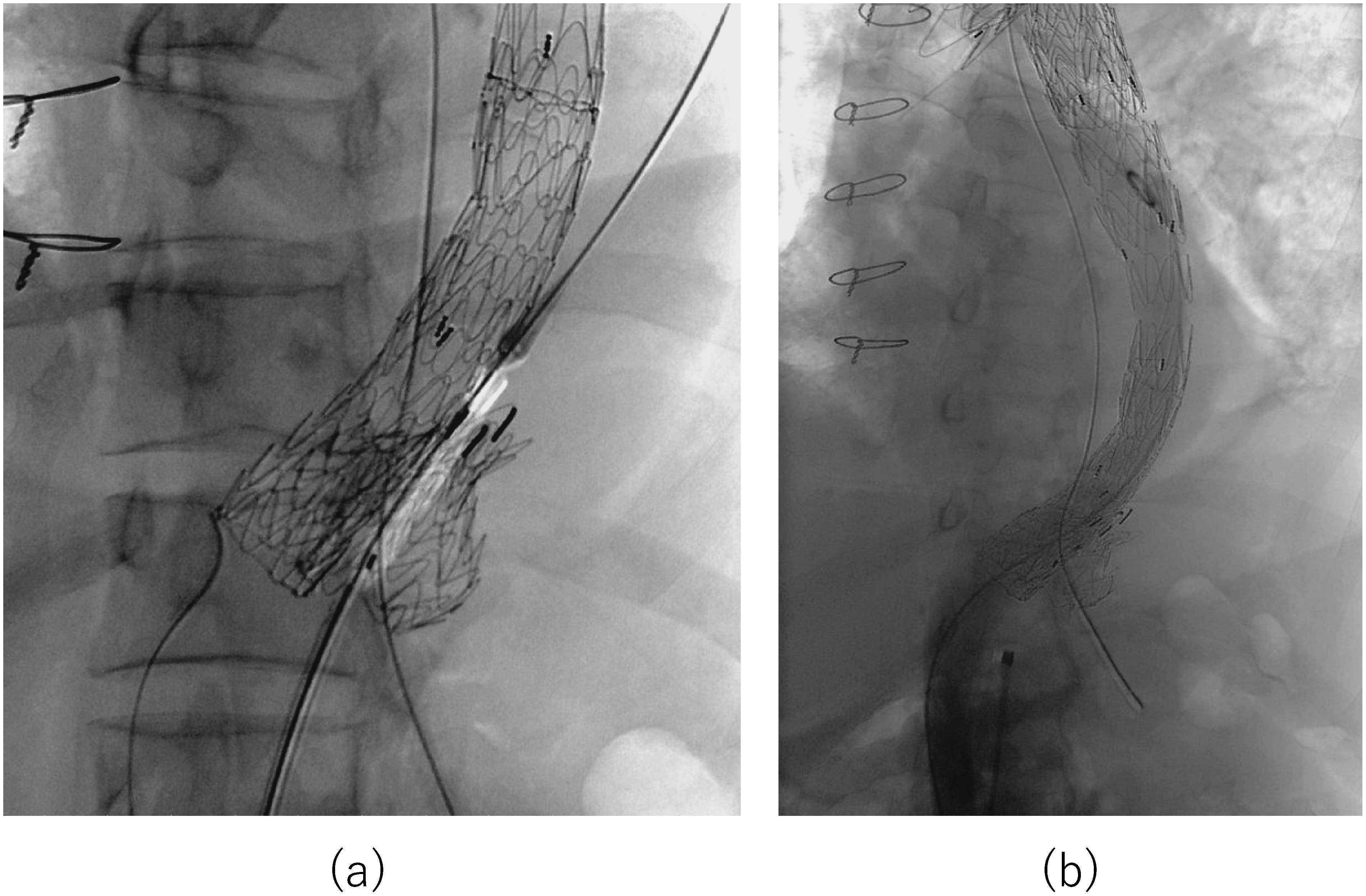
Fig. 3 TEVAR using the candy-plug technique. (**a**) The candy plug is placed in the FL and positioned parallel to the distal end of the stent graft in the TL. (**b**) Completion aortography shows marked decreasing retrograde flow into the FL.

TEVAR: thoracic endovascular aortic repair; FL: false lumen; TL: true lumen

The Ex-cuff was oversized by 10%–20% relative to the mean FL diameter at the level of candy-plug placement.^7)^ The stent graft or bare stent was also placed in the true lumen (TL) to prevent TL narrowing or flap injury (**Fig. 3**). The TL was covered when there were multiple minor entry tears originating from costal branches. Conversely, a short-length stent graft was placed parallel to the candy plug when there were no minor entry tears. The size of the stent graft was almost equal to the flap length where the stent graft was to be placed.

## Results

Technical success was achieved in six patients (86%), although TEVAR was successfully performed in all patients. In one patient, the candy plug was incorrectly positioned during deployment. A 36-mm Ex-cuff was used in 6 patients, whereas a 32-mm Ex-cuff was used in one patient, and a 16-mm AVP I was used in all patients.

No surgical complications were observed. **Table 2** shows perioperative outcomes within 30 days. Two patients required re-intervention. One patient underwent repeat TEVAR because of type Ia endoleak 28 days after the procedure. Another patient underwent repeat TEVAR because of a type III endoleak 14 days after the procedure. No other perioperative complications were observed. There were no endoleaks or complications at 6±2 months. There was no change in aneurysm size, although residual FL flow persisted in three patients (**Table 3**).

**Table 2:** Table 2 Perioperative outcomes

Case	Diagnosis	Maximum diameter (mm)	Mean FL diameter (mm)	Ex-cuff (mm)	Technical success	Complications	Re-intervention
1	cTBAD	58	27	32	Success	T1aEL	TEVAR
2	cTBAD	49	45	36	Success	None	None
3	Post type A	71	40	36	Failure	T3EL	TEVAR
	Post TEVAR						
4	Post type A rupture	44	34	36	Success	None	None
5	cTBAD	57	30	36	Success	None	None
6	cTBAD	68	34	36	Success	None	None
7	cTBAD	64	32	36	Success	None	None

cTBAD: chronic type B aortic dissection; FL: false lumen; Ex-cuff: Excluder aortic extender; T1aEL: type Ia endoleak; T3EL: type III endoleak; TEVAR: thoracic endovascular aortic repair

**Table 3:** Table 3 Clinical outcomes (6 months) (N=7)

Case	Endoleaks	FL flow	Size change	Complications	Re-intervention
1	None	−	No change	None	None
2	None	−	No change	None	None
3	None	＋	No change	None	None
4	None	−	No change	None	None
5	None	−	No change	None	None
6	None	＋	No change	None	None
7	None	＋	No change	None	None

FL: false lumen

Five patients underwent CECT 12±2 months postoperatively, and one patient (case 2) presented with a new ulcer-like projection requiring repeat TEVAR 399 days later. No candy-plug-related complications were observed. There was no change in aneurysm size in three patients. Aneurysm size decreased in two patients, whereas no cases of aneurysmal enlargement were observed (**Table 4**). The mean follow-up period was 593 days (range, 222–1225 days), and all patients were scheduled for a hospital visit.

**Table 4:** Table 4 Clinical outcomes (12 months) (N=5)

Case	Endoleaks	FL flow	Size change	Complications	Re-intervention
1	None	−	Shrink	None	None
2	None	＋	No change	New ULP	TEVAR
3	None	＋	No change	None	None
4	None	−	No change	None	None
6	None	＋	Shrink	None	None

FL: false lumen; ULP: ulcer-like projection; TEVAR: thoracic endovascular aortic repair

**Figure 4:**
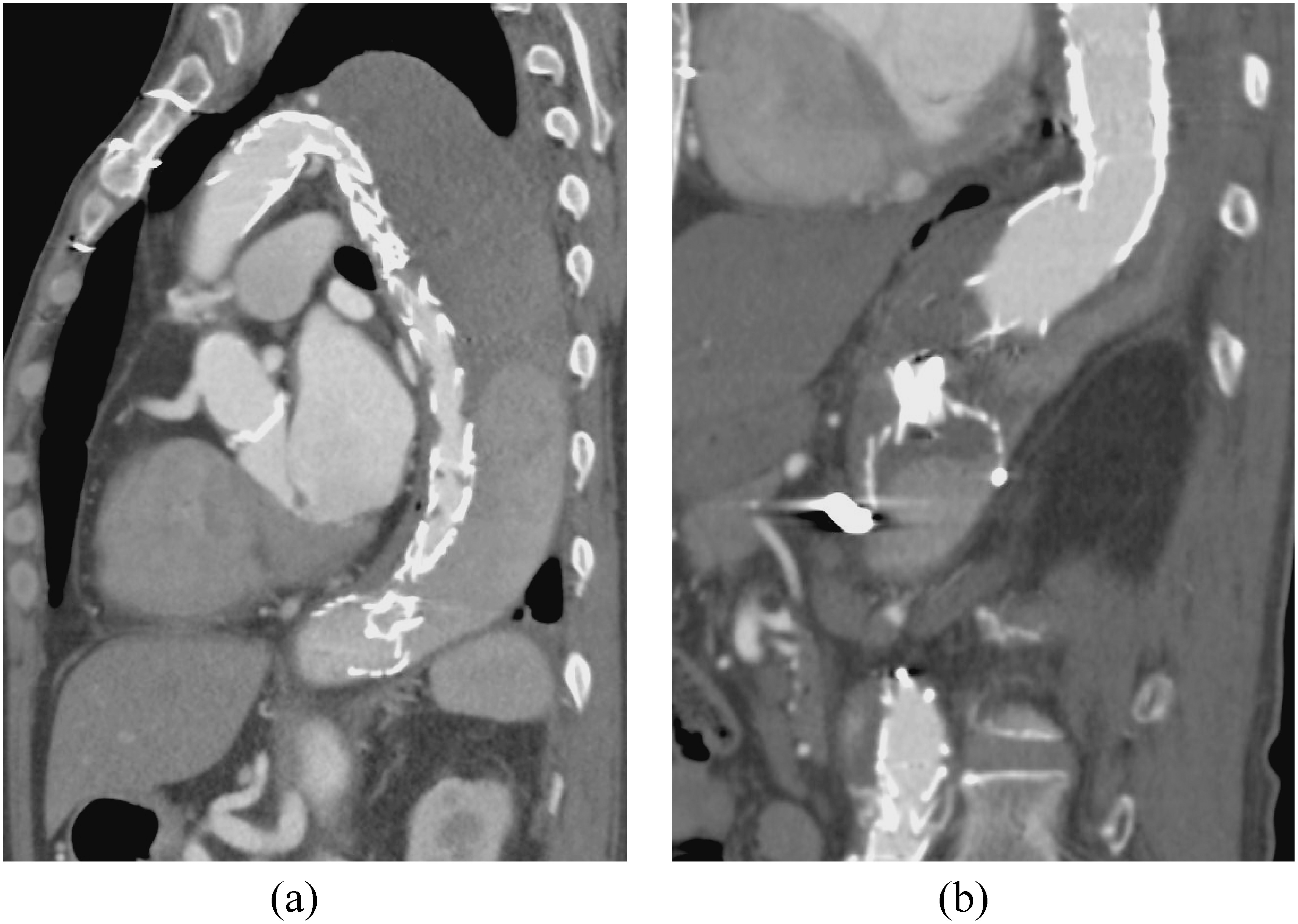
Fig. 4 Causes of residual FL flow. (**a**) CECT shows persistent retrograde flow though the AVP in the FL. (**b**) CECT shows persistent retrograde flow into the FL due to the migration of the candy plug.

FL: false lumen; CECT: contrast-enhanced computed tomography; AVP: Amplatzer Vascular Plug

**Figure 5:**
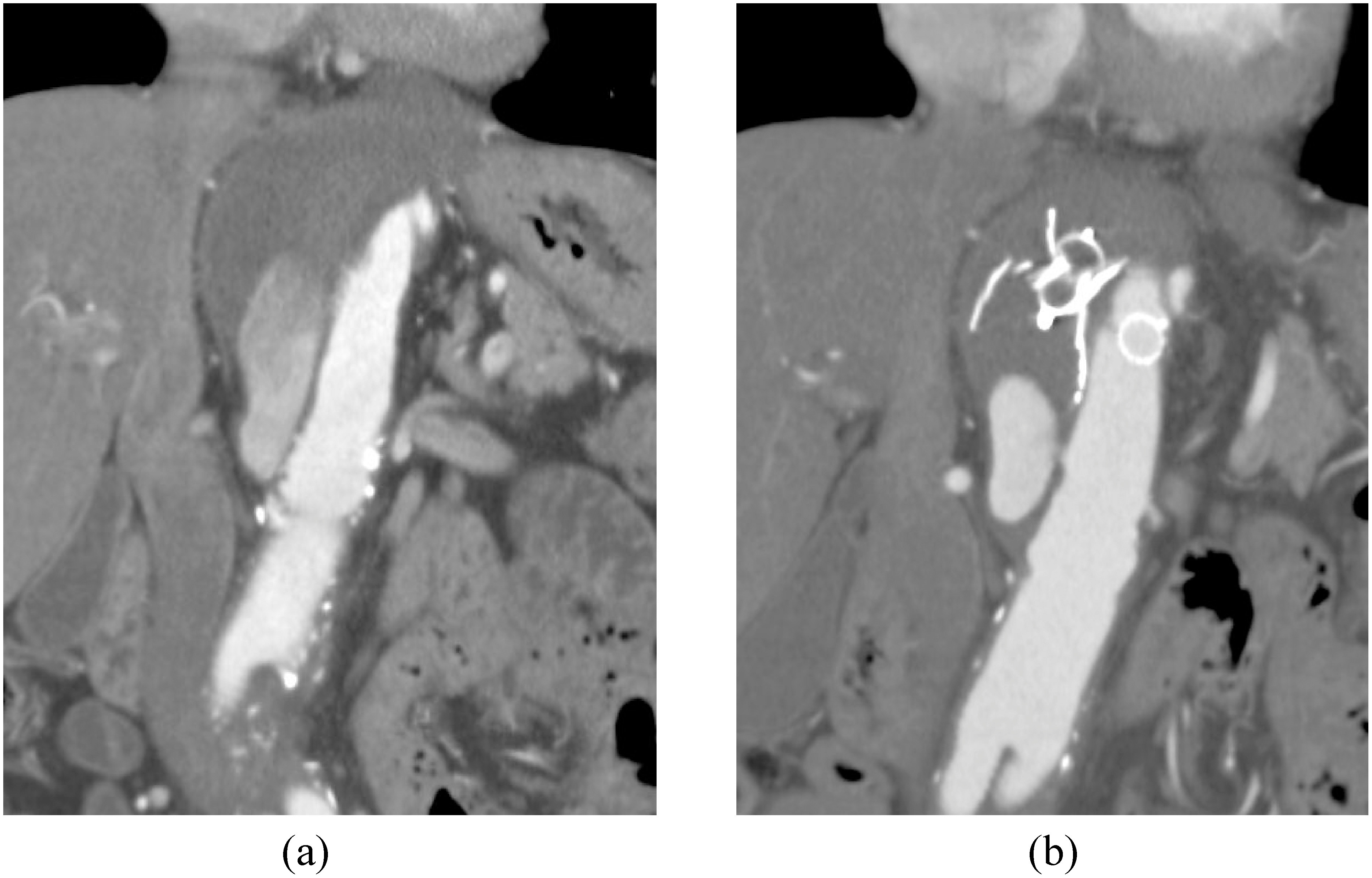
Fig. 5 Complete FL thrombosis despite placement of an undersized candy plug. (**a**) The mean FL diameter was 45 mm with a massive mural thrombus. The flow lumen measured 30 mm. (**b**) The 36-mm candy plug achieved complete FL thrombosis.

Total FL thrombosis was observed in 4 patients on CECT (clinical success rate, 57%) with a mean of 102 days (range, 10–222 days). Among the three patients with residual FL flow, two patients had incomplete AVP embolization (**Fig. 4a**) and one patient presented with candy-plug malposition (**Fig. 4b**). One patient had complete FL thrombosis using a 36-mm candy plug with a large FL of 45 mm and a mural thrombus (mean flow lumen diameter, 30 mm; **Fig. 5**).

FL: false lumen

**Figure 6:**
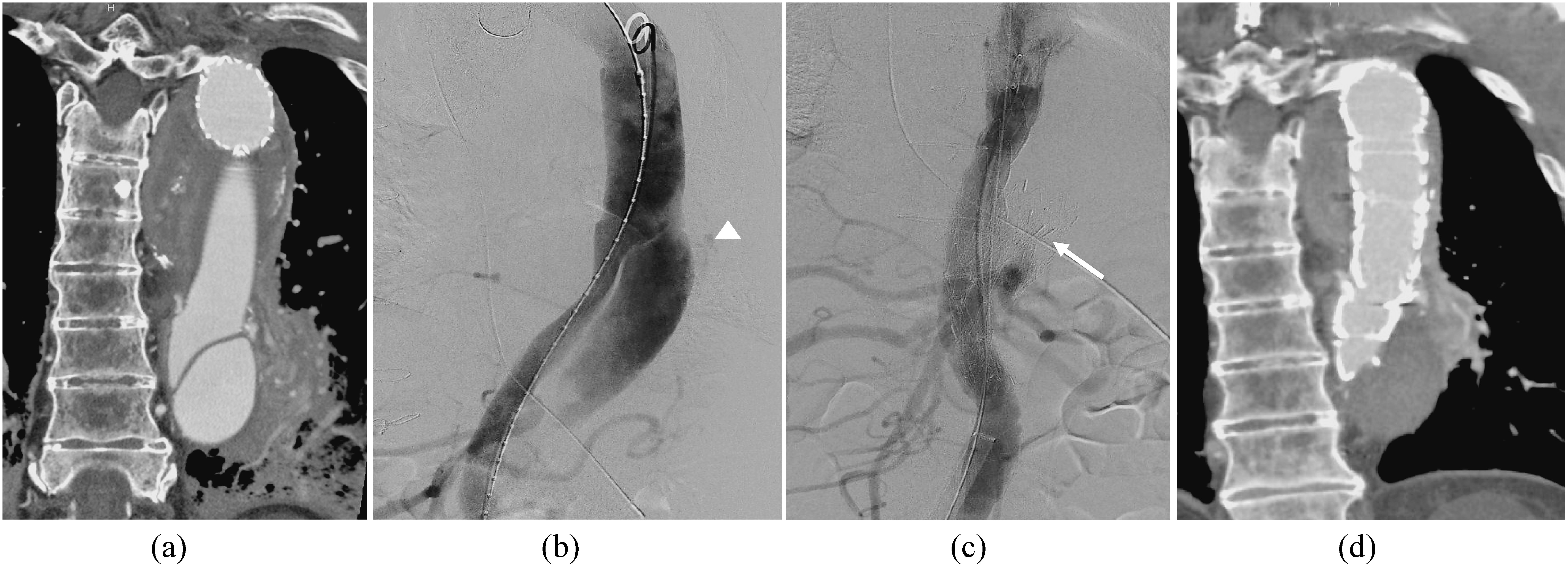
Fig. 6 A man in his 70s with hemoptysis after type A aortic dissection treated by total arch and descending aorta replacement and TEVAR. (**a**) Preoperative CECT shows hematoma around the anastomosis. (**b**) Aortography shows minor extravasation (arrowhead) close to the anastomosis and multiple distal entry points. (**c**) Postoperative TEVAR with the candy-plug technique shows no residual FL flow above the candy plug (arrow) on completion aortography. (**d**) Postoperative CT 10 days later shows FL thrombosis above the candy plug with a reduction in hematoma volume.

TEVAR: thoracic endovascular aortic repair; CECT: contrast-enhanced computed tomography; FL: false lumen

A man in his 70s with hemoptysis (case 4) presented with hematoma around the anastomosis without extravasation on preoperative CECT (**Fig. 6a**). The patient was scheduled for urgent TEVAR using the candy-plug technique 3 days later because his vital signs were stable without coagulopathy. The procedure was performed using a 22-F right femoral sheath (main access point) and a 20-F left femoral sheath for the candy plug. Aortography showed minor extravasation close to the anastomosis of the descending aortic graft (**Fig. 6b**). There was FL flow through multiple distal entry points at the level of the superior mesenteric artery, both renal arteries, and lower lumbar arteries.

First, a tapered 34-×30-×160-mm thoracic endograft (Valiant® Captivia®; Medtronic, Santa Rosa, CA, USA) was deployed just above the celiac trunk to cover the anastomosis. The FL was then cannulated through the lowest distal entry point via the left femoral sheath. The highest distal entry point was located at the level of the superior mesenteric artery (mean FL diameter, 34 mm), and the candy plug was deployed just above the superior mesenteric artery. We did not place the candy plug above the celiac trunk because of the large mean FL diameter of 38 mm.

A 26-mm Ex-cuff was deployed in the infrarenal TL (mean FL diameter, 25 mm) to close the 2 distal entry points. A 28-×95-mm modified aortic cuff (AFX; Endologix Inc., Irvine, CA, USA) was also placed between the thoracic graft and the aortic cuff in the TL to prevent TL narrowing after candy-plug deployment. The lowest entry point left to preserve left renal artery flow originated from the FL. Completion aortography showed no extravasation without any complications (**Fig. 6c**). Postoperative CT 10 days after the procedure revealed total FL thrombosis above the candy plug (**Fig. 6d**). The patient was discharged 12 days after the procedure and attended regular outpatient visits.

## Discussion

The Ex-cuff can be easily applied to the candy plug because, compared with the Zenith TX-2, it has a low profile and does not have to be unloaded from a delivery sheath.

This technique is useful, even for emergencies. However, in the present study, only 4 out of 7 patients (57%) achieved total FL thrombosis with a mean of 104 days. Although no FL enlargement was observed, one disadvantage of this technique is the time it takes to achieve FL thrombosis; therefore, this approach is not suitable for emergencies where rapid FL thrombosis is required. Rohlffs et al. reported 18 cases of custom-made candy plugs (Cook Medical) after their first report.^7)^ The candy-plug sizes used in Kölbel et al.’s study were 46 or 50 mm with an 18-mm midsection, and central occlusion was performed using a 22-mm AVP in 9 patients and a 20-mm Iliac ZIP Occluder (Cook Medical) in 9 patients. In their study, although the technical success rate was 100%, residual FL flow was seen in 3 patients during a mean follow-up period of 9 months. Among these patients, one died of FL rupture because of incomplete AVP embolization 5 months after the procedure, and an Iliac ZIP Occluder was recommended. In the present study, two of the three patients with a residual FL flow also had incomplete AVP embolization. The patient who presented with rupture had fortunately achieved FL thrombosis on CECT 10 days after the procedure. The possible explanation for this may be a marked decreased in FL flow due to the covering of multiple distal entry tears, which could aid AVP embolization; however, emergency situations may not permit the closure of multiple entry tears. Thus, the use of additional embolic materials, including coils or N-butyl-cyanoacrylate, should be considered for (impending) rupture. In the present study, unruptured aneurysm in six patients did not require additional embolization regardless of whether FL flow was present because no FL enlargement was demonstrated. Furthermore, one patient had a decrease in FL diameter despite residual FL flow.

The relationship between FL status and FL diameter remains unclear. However, if there is residual FL flow, careful management is mandatory, and early re-intervention should be considered when continuous FL enlargement is demonstrated.

Although our first case did not undergo stent-graft placement in the TL because of spinal cord ischemia,^6)^ all other cases underwent stent-graft placement to prevent TL narrowing or flap injury due to the candy plug.^8)^

When the candy plug was located below the celiac trunk, as it was in the case of rupture described above, stent-graft placement was not possible in the TL because visceral branches originate from the TL. Thus, bare-stent placement using the provisional extension to induce complete attachment technique is required.^9)^

Total FL thrombosis with an extensive TL covering can cause paraplegia. However, Rohlffs et al.^7)^ reported 1 case of transient paraplegia out of a total of 18 cases. The seven patients examined in this study did not present with transient paraplegia. Fenestrated/branched endovascular repair (EVAR) has a 10% risk of causing paraplegia^10,11)^ compared with the candy-plug technique, which preserves visceral branch perfusion. Furthermore, fenestrated/branched EVAR requires highly technical skill and can only be performed at a limited number of hospitals. Thus, the candy-plug technique has some advantages in terms of its safety and versatility.

Regarding the size selection of the candy plug, this technique depends on FL diameter because the maximum size of the Ex-cuff is 36 mm. Case 3 experienced candy-plug malposition during deployment because of poor attachment or instability caused by the use of an undersized Ex-cuff relative to the mean FL diameter of 40 mm.

The candy plug with thoracic stent-graft devices may be useful in cases of larger FL diameters. Marone et al.^4)^ reported a case of successful FL occlusion of ruptured aortic dissection due to residual distal entry points with a candy plug and a Zenith converter (Cook Medical) and an Iliac extension (Cook Medical) in a parallel fashion. Although the selection of appropriate size of each device and the possibility of vessel wall injury or TL narrowing are unclear, this parallel technique is a feasible option.

This study has several limitations. First, the study adopted a retrospective design and enrolled only a small number of patients. Second, the 4 patients with FL thrombosis only underwent plain CT from 1 year after the procedure; thus, the presence of endoleaks or recanalization was not clarified. Finally, the follow-up period was short; thus, long-term studies are needed to verify the findings presented.

## Conclusion

We conclude that the candy-plug technique using the Ex-cuff is technically feasible for FL occlusion because of its easy modification. However, it takes time to achieve AVP embolization, and close follow-up is mandatory when there is residual FL flow. Additional embolization should be considered in emergency cases.

The abstract of this manuscript was presented at the 23rd Annual Meeting of the Japanese Society of Endovascular Intervention in July 2017 in Nara, Japan.

## Notes

This is a translation of Jpn J Endovasc Interv 2018; 19: 29–35.
